# Epidemiology and Management Paradigm of Head and Neck Infections, Including COVID-19 Pandemic Period: A 10-Year Retrospective Study in a Maxillofacial Center of Cluj-Napoca

**DOI:** 10.3390/jcm13144046

**Published:** 2024-07-10

**Authors:** Cosmin Ioan Faur, Mădălina Anca Moldovan, Tino Paraschivescu, Sergiu Megieșan, Rareș Călin Roman

**Affiliations:** 1 Department of Oral Radiology, Iuliu Hatieganu University of Medicine and Pharmacy Cluj-Napoca, 400006 Cluj-Napoca, Romania; cosmin.faur@yahoo.com; 2 Department of Oral and Maxillofacial Surgery and Implantology, Iuliu Hatieganu University of Medicine and Pharmacy Cluj-Napoca, 400006 Cluj-Napoca, Romania; 3 Faculty of Dental Medicine, Iuliu Hatieganu University of Medicine and Pharmacy Cluj-Napoca, 400006 Cluj-Napoca, Romania; 4 Applied Mathematics, Imperial College London Alumni, London SW7 2AZ, UK; serg.flor99@gmail.com

**Keywords:** head and neck infections, odontogenic infections, deep cervical infection, epidemiology, COVID-19

## Abstract

**Background.** The management of odontogenic infections varies across the globe. To shed light on the subject, this study delves into the practices of the Oral and Maxillofacial Department at Cluj-Napoca County Hospital. **Material and Methods.** This comprehensive retrospective analysis of 10 years of clinical experience covered a range of factors, including demographics, clinical and investigations factors, medical and surgical treatment approaches, and follow-up. Additionally, the study examined the change in trends over the course of the COVID-19 pandemic. **Results.** While head and neck infection occurrence had a decreasing trend before the COVID-19 pandemic, during the pandemic era the number of patients almost doubled. The infections were prevalent in the submandibular space, teeth being the prevalent cause. Young adults are prone to suffer from odontogenic infections. High levels of C reactive protein, ASA II-IV risk, and hepatic dysfunction indicated a longer time of hospitalization. The majority of antibiograms were negative, and the positive ones indicated Streptococcus and Staphylococcus species as prevalent pathogens. Extra- or intraoral surgical drainage and wide-spectrum antibiotics proved to be the workhorse for odontogenic infections. **Conclusion.** This study advances our understanding of oral and maxillofacial surgery and offers actionable strategies for improving patient outcomes in similar healthcare institutions worldwide.

## 1. Introduction

Head and neck infections are caused by various pathologies, such as infected lymphadenopathy or salivary glands, tumors, or trauma, but the most common origin is in dental decay complications, such as periapical abscesses [1]. The direction of pus spreading through alveolar bone in odontogenic infections is dictated by the less resistant bone density with further exteriorization into a peri-osseous space, and next to a fascial plane [2]. In non-compliant patients who delay to address medical care or immunosuppressed patients, as well as patients with healthcare problems, such as diabetes mellitus, the infection spreads to adjacent spaces and it can be life-threatening [3]. The incidence of dental-related abscesses in Europe has declined in recent years [4]. However, during the COVID-19 pandemic, the incidence of head and neck infections showed a changed trend, with an increased number of patients being admitted to hospitals for dental-related infections [5].

Celsius signs, asymmetry, and pain are the classic signs of pus presence. Trismus, dysphagia, and fever indicate deep cervical spaces’ involvement [6]. In addition to the patients’ complaints and clinical signs, the diagnosis of head and neck infections is based on imaging. Orthopantomography (OPG), computed tomography (CT), and cone-beam computed tomography (CBCT) assist the specialist in localizing the pus and the starting point of infection [7]. The submandibular space is the most common situs for head and neck infection, with the second and third molar being the most frequent cause [8]. Usually, mixed bacterial flora is identified in these infections. Also, streptococcus species are frequently seen in pus culture [3,9]. Surgical treatment by drainage in conjunction with antibiotic administration is currently the state of the art in head and neck infection management. The incision for drainage can be done intra- or extraorally, or a combination, followed by tube insertion for lavage with antiseptic substances [10].

Currently, there is limited knowledge regarding the epidemiology of head and neck infections in Romania, and recommended protocols for such cases are not available. With this in mind, our objective was to shed light on the prevalence and epidemiological characteristics of head and neck infections at the Oral and Maxillofacial Department at Cluj-Napoca County Hospital. Additionally, we have sought to assess the center’s internal protocol for managing infections and examine any changes that may have arisen in response to the COVID-19 pandemic.

## 2. Materials and Methods

This study included patients who were admitted to the Department of Maxillofacial Surgery, Cluj-Napoca County Hospital, Romania between January 2013 and December 2022, and who were diagnosed with head and neck infections through clinical and radiological examination. Patients with radiation-related infections, medication-related osteonecrosis of the jaws, necrotizing fasciitis, or ear–nose–throat infections, as well as patients with insufficient data from the discharge document, were excluded from the study.

The Cluj-Napoca County Hospital’s informatic system (AtlasMed) was used to retrospectively gather patient data, including demographic, clinical, radiological, and treatment information, which were then anonymized. Patients were divided into four categories based on age: children (0–18 years old), young adults (19–35 years old), mature adults (36–60 years old), and elderly patients (over 61 years old). All patients underwent an intraoral, extraoral, or combined intra–extraoral incision for pus drainage, which was performed using sedation, local or general anesthesia, and antibiotic therapy, either as monotherapy or in combination, based on the antibiogram result. Patients who did not have a radiological exam prior to admission were evaluated using 2D (orthopantomography) or 3D (spiral CT or CBCT) exams. Infections were classified as peri-osseous (buccal, palatal, or body of the mandible spaces), primary fascial plane (submental, submandibular, sublingual, buccal spaces), second fascial plane (temporal, masseter, pterygomandibular, retropharyngeal, prevertebral, parotid spaces), and diffuse (Ludwig angina and hemifacial phlegmon), and pus was harvested using specialized pus collectors with and without wet medium. The patients’ health status was assessed using the American Society of Anesthesiologists scale [11]. A questionnaire was administered to the patients to evaluate their symptoms, which were further classified as mild, moderate, and severe.

The statistical analysis was performed using Python 3.10 (Jupyter Notebook, New York, NY, USA) Software and the following techniques: statistical tests (Kolmogorov–Smirnov, Kruskall–Wallis, Student *t*-test, Pearson’s correlation coefficient), trend visualization (linear regression), pie charts, bar plots, correlation matrices, distribution matrices, and run plots. *p* < 0.05 was set as the statistical significance threshold.

This study was approved by the Ethics Committee of “Iuliu Hatieganu” University of Medicine and Pharmacy (No 73/06.03.2026), and it is in accordance with the updated Declaration of Helsinki.

## 3. Results

A total of 150 COVID-19-negative patients suffering from head and neck infections were included in this study, of which 115 responded to the questionnaire. The differences between pre- and pandemic patients’ groups can be seen in Table 1. One 78-year-old male patient, suffering from Ludwig angina, died after 30 days of hospitalization.

**Figure 1:**
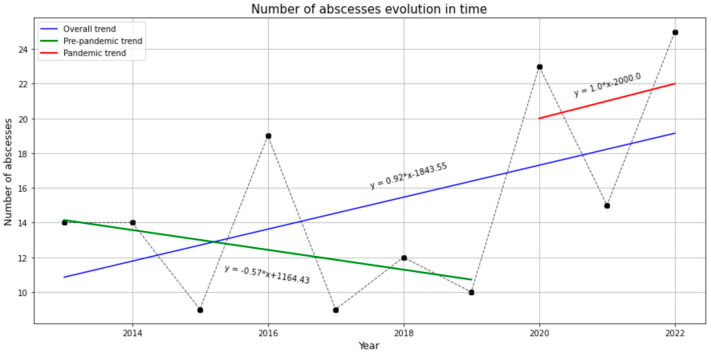
The evolution of head and neck infections over time.

The number of head and neck infections decreased between 2013 and 2019. However, during the pandemic we observed an increase in patients’ admission to the hospital due to head and neck infections. The changes can be seen in Figure 1.

The head and neck infections affected mostly young adults, with the peak in the second and third decades of life. There was no statistical difference between pre- and pandemic eras regarding age. The male-to-female ratio was 1:1.2, and no difference was observed between places of living. However, the level-of-education evaluation indicated that 47% of the patients studied through primary school and 8% of the patients admitted to the hospital for head and neck infections underwent university studies. Also, a significant number of patients had no healthcare insurance, with approximately 24% of the active population aged between the second and sixth decade of life being unemployed.

The head and neck infections were mostly located in a primary fascial space, the submandibular space being the predominantly involved situs with 55% of the cases (82 patients), alone or in conjunction with other adjacent sites. Teeth were the most common cause of head and neck infection, and abscesses related to decay complication or post-extraction socket represented 94% of the patients. One young patient (1 year old) had a salivary-gland-related abscess; the rest of the tumor-, trauma-, or salivary-gland-related infections were present in mature adults or elderly patients (nine patients).

Out of the 150 patients examined, 122 patients (81%) underwent radiological examination when they were admitted to the hospital. The remaining patients (19%) had already undergone X-ray exposure and presented to the doctor with mild symptoms. The most commonly used radiological examination was orthopantomography (OPG). However, in cases where abscess involvement was more complex, a 3D examination or a combination of OPG and CT was preferred. It has been observed that access to 3D examination has increased significantly since 2018, with 26 patients undergoing the 3D examination as compared to 7 patients in the earlier period (*p* < 0.05).

The classic symptoms and signs of head and neck abscess represented by pain, asymmetry, and Celsius signs were referred to by every patient and considered as mild symptomatology if there were no other complaints. Moderate symptomatology, such as trismus and dysphagia, was observed in 14 primary fascial planes, 3 peri-osseous abscesses, 4 secondary fascial planes, and 3 diffuse infections. On the other hand, severe symptoms, such as dyspnea and retrosternal pain, were observed only in the diffuse infection patients (two patients). Fever was observed in 43 patients, most of them complaining of moderate symptomatology (90%). Fever was most commonly present in children (39% of children) and young adults (31% of young adults), compared to mature adults (23%) and senior patients (15%).

The blood tests usually indicated elevated levels of inflammatory markers, such as C-reactive protein (CRP), fibrinogen, and leukocytes. The CRP and fibrinogen were the most sensitive markers that highlighted the intensity of the systemic response to the infection. Males and females had similar values of CRP and fibrinogen. The CRP and fibrinogen levels can be seen in Table 2. The elevated levels of CRP (>5 mg/dL) were statistically correlated with a longer hospitalization (Pearson value = 0.3; *p* > 0.01). Different from inflammatory markers, we observed other blood test changes in 65 patients, such as glucose levels, hepatic or renal dysfunction (ALAT, ASAT, creatinine, urea, etc.), and metabolism changes (e.g., cholesterol level). We quantified these changes as well as the health status in ASA risk (Table 1). However, most of the patients presented no significant health problems, with 43% being classified as II–IV ASA risk. Diabetes mellitus was present in 42 patients (28%), most of them having submandibular-space abscesses (30 patients) and staying 3 (2,3) days in the hospital. The hepatic-dysfunction patients accounted for 28 cases (18%) and statistically stayed a longer time in the hospital compared with patients without any liver dysfunction (*p* < 0.01). However, the patients classified in ASA II–IV had a prolonged hospitalization compared with ASA I. (*p* < 0.05)

**Figure 2:**
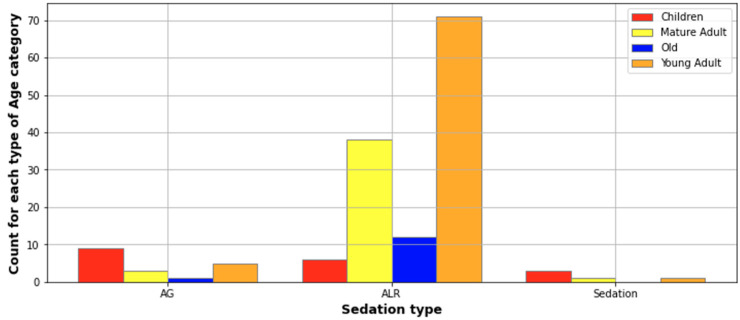
Description of patients that underwent anesthesia as related to age.

**Figure 3:**
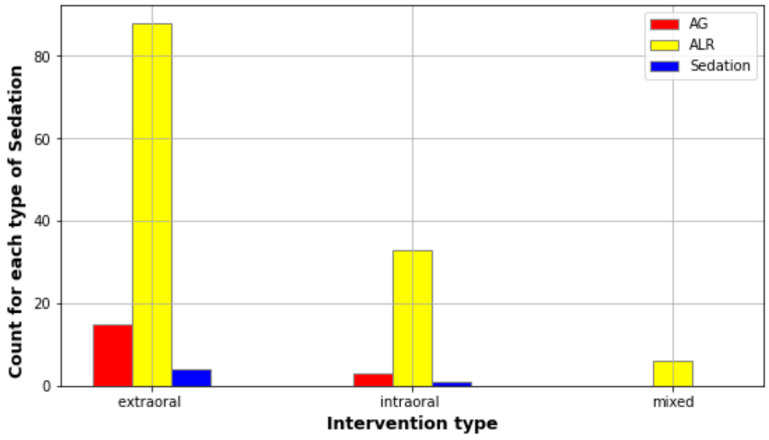
Description of the correlation between type of anesthesia and type of drainage.

Surgical drainage was performed in all admitted patients, and tracheostomy was necessary in two patients. General anesthesia or sedation was used for 19 patients who underwent extraoral or mixed incisions. Most of those patients, namely 10 of them (52%), were aged under 18 years old (Figure 2). Also, intraoral incisions were performed under general anesthesia or sedation in two cases, the patients being under 18 years old. Twelve patients needed a second surgery (seven extraoral, three intraoral, and two associated incisions), three of these being aged under 18 years old (Figure 3). Reintervention was seen in 13 patients, with children being most likely to suffer a second surgery (16.6% of the children). The patients who required reintervention stayed longer in the hospital (6.3 ± 2.2 days) compared with patients with only one surgery (4.52 days ± 1.84, Pearson correlation = 0.2, *p* < 0.001).

The tubes were removed from the second day after surgery to 30 days, with a mean ± standard deviation of 3.5 ± 1.5 days. They were removed after a longer time (more than 5 days) in mature adults (5 to 6 days in 53% and more than 7 days in 4% of the mature adults) and older patients (5 to 6 days 60% and more than 7 days for 30% of the elderly patients) comparative with children (5 to 6 days 23% and more than 7 days for 38% of the mature adults) and young adults (5 to 6 days 30% and more than 7 days for 17% of the young adults). After cessation of the infection and symptomatology, 28 patients (18%) were referred for conservative treatment of the causal teeth, the rest of them being extracted from the study.

The results of antibiogram from pus that was harvested from the infection situs were mostly negative (75% of patients). Commonly, streptococcus (16 patients) and staphylococcus (13 patients) were identified in the positive antibiograms, the rest being pathogens, such as enterococcus (4 patients) or others less frequent (e.g., Proteus in 1 patient). The positive antibiograms were mostly identified in fascial plane infections (63%patients), in comparison to peri-osseous infections (11% of patients) where the antibiogram was mostly negative (*p* < 0.01). Streptococcus and staphylococcus species were prevalent in primary-space abscesses (21 patients) and other pathogens in secondary spaces (4 patients). Also, streptococcus and staphylococcus species induced increased levels of CRP (>5 mg/dL in 13 patients) and fibrinogen (>400 mg/dL in 13 patients).

The antibiotic regimen was administered based on the symptoms, ASA risk, and location/extension of the abscess. Except for the 34 patients with peri-osseous abscesses, for which antibiotic monotherapy was administrated, the fascial-plane abscesses received the association between a beta-lactam or cephalosporin (A), gentamycin (G), and anaerobic antibiotic (M) (clindamycin or metronidazole). Further, if the antibiogram was negative, the patients continued treatment with the triple association, A + G + M (111 patients). Otherwise, the regimen was adjusted according to the antibiotic sensitivity of the pathogen or continued with only one sensitive antibiotic from the triple-association initial regime. Second-line antibiotics like tazobactam, vancomycin, and ciprofloxacin were administered during hospitalization as a change of strategy in case of multi-drug resistance of the identified pathogens, as seen in five patients according to the antibiogram results.

**Figure 4:**
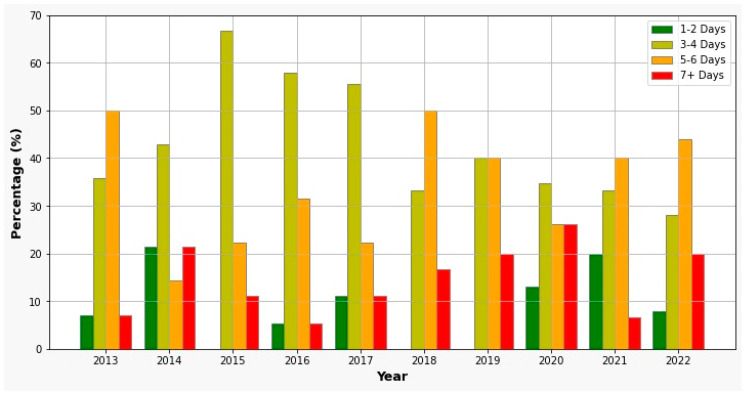
Hospitalization days trend.

The length of hospital stays has increased from an average of three days to four days in recent years (Figure 4). Patients who had fascial-plane involvement required a longer hospital stay (5.0 ± 1.8 days) compared to those with peri-osseous infections (3.3 ± 1.6 days), with a significant difference (*p* < 0.01). Additionally, patients who needed extraoral or associated incisions (4.9 ± 1.8 days) required more specialized treatment and stayed longer in the hospital than those who received intraoral incisions (3.7 ± 2.1 days), with a significant difference (*p* < 0.01). Patients who received the triple association of antibiotics or second-line antibiotics also had a prolonged hospital stay (4.85 ± 1.9 days), compared to those who received monotherapy (3.82 ± 1.5 days).

## 4. Discussion

To our knowledge, this study is the first to report on the epidemiology of head and neck infections in Romania and the treatment protocol used in a National Healthcare System Hospital. During the COVID-19 pandemic, there was an increased prevalence of dental infections, an alteration from the reduced infection trend in the pre-pandemic era [12]. The pandemic period was a difficult time due to lockdowns that reduced access to medical care for non-urgent, non-COVID-19 patients [5,13]. In the lockdown periods, dental offices were either closed or the additional anti-COVID-19-infection measures, such as additional protection equipment or increased time for office disinfection, increased the costs and reduced the number of patients that could be treated [14]. As a result, decays were not treated, and complications like dental abscesses occurred more frequently [15]. Our center experienced approximately twice the prevalence of dental-related infections during the COVID-19 pandemic compared to the pre-pandemic period. However, demographic features remained unchanged.

The most abscesses occurred in young adult patients, with no difference with regard to sex and place of living. This is consistent with other research sources that indicate that the younger population is at higher risk due to tooth loss at older ages [16]. Also, different from another study, no difference in the male-to-female ratio or in the place of living between cohorts was observed [17]. Our findings can be related to economic circumstances and education level. Almost one-quarter of the adults had no healthcare insurance, which is guaranteed by the National Healthcare system for all professional active persons, hence almost one-quarter of adults that were hospitalized for head and neck infections were unemployed. First, the lack of a national program for primary dental assistance and screening, especially in young patients with decay problems, may contribute to the negligence of oral health. The nine mature adults and elderly patients (16% of patients aged >35 years old) had no dental cause of the abscess, compared with younger patients (0.01% of patients aged <35 years old). Second, most of the hospitalized patients were adults who had attended only primary school.

Patients with associated healthcare problems, such as diabetes mellitus, hepatic or renal dysfunction, immunosuppression, or immune dysfunction are at risk of developing infections, frequently dental-related infections [18]. More than half of the patients had no medical history and were classified as ASA I risk, indicating that abscesses appeared despite the medical conditions. However, diabetes mellitus and hepatic dysfunction, which were present in 28% and 18% of the patients, respectively, were associated with prolonged hospitalization due to the difficult management of the infection. Also, the CRP and fibrinogen of these patients were highly increased compared with ASA I risk patients. In terms of sex, we observed no difference in immune reaction to infection (CRP, fibrinogen). Generally, the high levels of CRP and fibrinogen indicated a more intense infection associated with prolonged hospitalization [17,19]. Even though advanced infection with deep fascial-plane involvement was present in 116 patients, most of the patients included in this study indicated mild symptomatology (124 patients). In contrast to severe symptomatology, fever primarily appeared in patients who experienced moderate symptoms and in children who exhibited a distinct response to infections relative to adult organisms.

Similar to other research, submandibular-space abscess was the most common infection situs of hospitalized patients [20]. Generally, the lower molars are the most frequent cause of submandibular-space abscess [21]. The hospitalized patients were examined in the majority of cases by OPG (59%), which was enough to detect the causal tooth. A 3D examination, such as CT or CBCT, was used to identify the involvement of adjacent fascial planes or the diffuse infection. Moreover, access to 3D examination has increased since 2018, and in the COVID-19 pandemic, CT or CBCT was more commonly used compared with the pre-COVID era. Although oral and maxillofacial surgeons have worked successfully with panoramic radiography, the limitations of this imaging technique include variable magnification, distortion, superimposition of structures, and suboptimal imaging of structures that are not in the focal trough. CBCT has overcome these limitations. Depending on the field of view, this method projects a large area of the facial skeleton beyond the limits of a 2D X-ray/panoramic radiograph. Because CBCT slices can be reformatted and viewed in a variety of orientations, anatomical structures are not superimposed. Over the past decade, the technology and design of CBCT scanning equipment have made this physically and financially possible [22].

Since the discovery of antibiotics, the mortality of head and neck infection patients radically decreased [23]. In our study, we identified only one elderly patient who has not survived, due to Ludwig angina, with a lower first molar causal tooth. This patient presented several medical conditions, such as diabetes mellitus and hypertension, and developed a systemic infection.

The drainage of the pus was the first treatment of the abscesses and remained the gold standard [17]. The extraoral or mixed incisions were the most frequently used procedures (76% of the patients), being used mostly for fascial-plane infections. Intraoral incisions, on the other hand, were used in the majority of cases for the peri-osseous abscesses and in a few cases for the fascial planes’ abscesses with an intraoral evolution (e.g., submandibular abscess with reccesus evolution). These findings are similar to other research data [12]. Adjuvant to the drainage, tube insertion in the pus cavity is mandatory to keep a patent passage for pus evacuation and for periodic lavages [24]. The anesthesia in most patients was local, except in children and for deep fascial planes, for which sedation or general anesthesia was used, in order to provide a more comfortable surgery for the patients. However, reintervention was needed more frequently in children due to the cooperation difficulty for daily lavages and the definitive treatment. In mature adults, only nine needed reinterventions, for adjusting the tubes’ location and debridement.

Nowadays, there is a concept of antibiotic-use de-escalation due to antibiotic abuse and increasing pathogen resistance [25]. However, the antibiotic association is crucial in head and neck infection treatment. Due to the high number of non-diagnosed antibiograms, the coverage of a large spectrum of pathogens was required, and we administered an association of a beta-lactam, gentamycin, and anaerobic target antibiotic [26]. The non-diagnosis antibiogram may be due to the harvesting procedures, the transport, or the highly sensitive pathogens that do not survive ex vivo (112 samples) [27]. Further, for the positive antibiogram, which detected mostly staphylococcus and streptococcus species, we de-escalated the antibiotic therapy to only one product to which the pathogen was sensitive. We had to use the second-intention antibiotic therapy in 3% (five patients) due to the highly drug-resistant pathogens, such as MRSA or Proteus species.

The tubes that were inserted into the abscess space for antiseptic lavages are kept in place as long as the pus is drained, usually two to three days [28]. However, the tubes remain longer for deep fascial planes in mature and elderly patients, who had more difficult-to-treat infections. Further, if the infection was not severe, the abscess was located in a peri-osseous space, and the tooth could be preserved and had a prosthetic value, the endodontic treatment was performed. Only 18% of the patients met all these conditions, were compliant, and benefited from the conservative treatment; the rest were radically treated by extractions.

We observed an increased trend of longer hospitalization since 2018. This could be explained not by more severe infections of the patients, but by more intense care of the patients and a different protocol in which surveillance of the patient’s antibiotic treatment was performed. We also observed longer hospitalization of the patients with advanced fascial-plane involvement and more medical conditions associated with the infections. However, the association between extraoral or mixed incisions with triple-antibiotic association proved to be a valuable treatment protocol due to the reduced hospitalization compared with monotherapy, especially in non-diagnosis antibiogram patients.

The present study has certain limitations that may interfere with the statistical analysis’s accuracy. One such limitation is the relatively small number of patients included in the study, which could pose challenges in terms of obtaining statistically significant results. We included only COVID-19-negative patients that were admitted in our department, the COVID-19 patients being treated for head and neck infections in specialized COVID-19 centers. Additionally, the high number of non-diagnosed antibiogram samples could also impact the analysis, as these samples may not provide a complete picture of the microorganisms involved or the antibiotic resistance patterns in the patient population being studied. While efforts were made to mitigate these limitations, it is important to remember them when interpreting the study’s findings. This study may offer a protocol for head and neck infection treatment that needs to be validated by prospective research.

## 5. Conclusions

In conclusion, the intra- or extraoral incisions for drainage of pus collection need to be adjusted considering the location of the abscess and medical status of the patient. While most surgical drainages can be performed using local anesthesia, general anesthesia or sedation is mandatory in children and for deep fascial involvement. Large-spectrum antibiotic association must be considered as a first line immediately after admission to the hospital and in patients with non-diagnosis antibiogram, but the antibiotic therapy must be quickly adjusted after a positive microbiologic examination result, in accordance with the antibiogram. Conservative treatment of the causal tooth is suitable in less severe, peri-osseous infections with a tooth that could be preserved and has a prosthetic value. The COVID-19 pandemic reversed the descending trend of head and neck infections’ prevalence, with almost doubled hospitalization during the pandemic era.

## Figures and Tables

**Table 1:** Patients’ demographic, clinical, radiological investigation, and treatment features.

Selected Parameters (*n* = Patients (%))		2013–2022	Pre-Pandemic	COVID-19 Pandemic	Statistical Analysis
Number of patients (mean/year)		150 (15)	87 (12)	63 (21)	*p* < 0.01
Age (mean ± standard deviation)		34 ± 19 years	33 ± 19 years	34 ± 18 years	*p* > 0.05
Sex	Male	81 (54%)	45 (52%)	36 (57%)	*p* > 0.05
	Female	69 (46%)	42 (48%)	27 (43%)	
Living place	Urban	78(52%)	47 (54%)	32 (51%)	*p* > 0.05
	Rural	72 (48%)	40 (46%)	31 (49%)	
Education	Primary school	71 (47%)	41 (47%)	30 (48%)	*p* > 0.05
	Gymnasium	12 (8%)	8 (9%)	4 (6%)	
	High school	14 (8%)	8 (9%)	6 (10%)	
	Technical school	7 (5%)	2 (3%)	5 (2%)	
	University	12 (8%)	7 (5%)	5 (4%)	
Health insurance	Yes	128 (85%)	70 (80%)	58 (92%)	*p* > 0.05
	No	22(15%)	17 (20%)	5 (8%)	
ASA risk	I	85 (57%)	54 (62%)	31 (49%)	*p* > 0.05
	II	50 (33%)	25 (28%)	25 (40%)	
	III	11 (7%)	4 (5%)	7 (11%)	
	IV	4 (3%)	4 (5%)	0 (0%)	
Localization	Peri-osseous	34 (23%)	20 (23%)	14 (22%)	*p* > 0.05
	Primary space	94 (63%)	56 (64%)	38 (60%)	
	Secondary space	17 (11%)	8 (9%)	9 (14%)	
	Diffuse infection	5 (3%)	3 (3%)	2 (3%)	
Hospitalization days (mean ± standard deviation)		4.7 ± 1.9	4.5 ± 1.7	5 ± 2.2	*p* < 0.01
Imaging	2D radiograph (orthopantomography)	89 (59%)	59 (68%)	30 (48%)	*p* > 0.05
	3D imaging (cone-beam computed tomography or spiral computed tomography)	22 (15%)	9 (10%)	13 (21%)	
	2D + 3D	11 (7%)	3 (3%)	8 (13%)	
	Imaging prior to hospitalization performed in another center	28 (19%)	16 (18%)	12 (19%)	
Cause of the abscess	Dental	134 (89%)	77 (89%)	57 (90%)	*p* > 0.05
	Trauma	2 (1%)	2 (2%)	0 (0%)	
	After tooth extraction	8 (5%)	4 (5%)	4 (6%)	
	Infected tumor or adenopathy	2 (1%)	1 (1%)	1 (2%)	
	Salivary glands	4 (3%)	3 (3%)	1 (2%)	
Symptoms	Mild	124 (83%)	82 (94%)	42 (67%)	*p* < 0.05
	Moderate	24 (16%)	4 (5%)	20 (32%)	
	Severe	2 (1%)	1 (1%)	1 (1%)	
Fever	Yes	43 (29%)	34 (39%)	9 (14%)	*p* < 0.05
	No	107 (71%)	53 (61%)	54 (86%)	
Type of incision	Intraoral	36 (24%)	18 (21%)	18 (29%)	*p* > 0.05
	Extraoral	107 (71%)	66 (76%)	41 (65%)	
	Mixed	7 (5%)	3 (3%)	4 (6%)	
Type of anesthesia	Local	127 (85%)	75 (86%)	52 (83%)	*p* > 0.05
	Sedation	5 (3%)	2 (2%)	3 (5%)	
	General	18 (12%)	10 (11%)	8 (13%)	
Types of germs that caused infection	Staphylococci	13 (9%)	9 (10%)	4 (6%)	*p* > 0.05
	Streptococci	16 (10%)	7 (7%)	9 (14%)	
	Enterococci	4 (3%)	1 (1%)	3 (5%)	
	Others	6 (4%)	2 (2%)	3 (5%)	
	Non-diagnostic	111 (73%)	69 (79%)	44 (70%)	
Antibiotic therapy	Aminopenicillin monotherapy	25 (17%)	18 (21%)	7 (11%)	*p* > 0.05
	Cephalosporin monotherapy	8 (5%)	4 (5%)	4 (6%)	
	Clindamycin monotherapy	1 (1%)	1 (1%)	0 (0%)	
	Aminopenicillin + gentamicin + anaerobic antibiotic (clindamycin or metronidazole)	70 (47%)	41 (47%)	29 (46%)	
	Cephalosporin + gentamicin + anaerobic antibiotic (clindamycin or metronidazole)	41 (27%)	21 (24%)	20 (32%)	
	Other (similar to antibiogram, for which previous antibiotic regime was resistant)	5 (3%)	2 (2%)	3 (5%)	
Antalgic treatment	Minor	46 (31%)	30 (34%)	16 (25%)	*p* > 0.05
	Moderate	100 (67%)	55 (63%)	45 (71%)	
	Advanced	4 (2%)	2 (2%)	2 (3%)	

**Table 2:** C-reactive protein, fibrinogen, and leukocyte values.

Inflammatory Markers	Values	2013–2022	Pre-COVID	COVID	Statistical Analysis Pre- vs. COVID-19 Pandemic
C-reactive protein	<5	23 (31%)	6 (26%)	17 (33%)	*p* > 0.05
	5–10	15 (20%)	8 (35%)	7 (14%)	
	>10	36 (49%)	9 (39%)	27 (53%)	
Fibrinogen	<400	13 (18%)	3 (11%)	10 (22%)	*p* > 0.05
	400–600	31 (42%)	13 (48%)	18 (39%)	
	>600	29 (40%)	11 (41%)	18 (39%)	
Leukocytes	<15.000	36 (67%)	15 (68%)	21 (66%)	*p* > 0.05
	15–20.000	14 (26%)	3 (14%)	11 (34%)	
	>20.000	4 (7%)	4 (18%)	0 (0%)	

## Data Availability

The data presented in this study are available on request from the corresponding author due to data confidentiality of patients.
